# Supplementary material for: Sandwich Fluorescence Detection of Foodborne Pathogen Staphylococcus aureus with CD Fluorescence Signal Amplification in Food Samples
Source: Foods. 2022 Mar 25;11(7):945. doi: 10.3390/foods11070945 (PMC8997861; doi:10.3390/foods11070945)
Supplement: Supplementary file 1 [file foods-11-00945-s001.zip › foods-1611256-Supplementary Materials.pdf]

foods

Supporting Information

Sandwich fluorescence detection of foodborne pathogen *Staphylococcus aureus* with CD fluorescence signal amplification in food samples

Han Du [1,2], Tao Ping[2], Wei Wu [2,3] and Qingli Yang [1,2,*]

[1] College of Food Science and Engineering, Shandong Agricultural University, Taian, Shandong, China
[2] College of Food Science and Engineering, Qingdao Agricultural University, Qingdao, China
[3] Qingdao Institute of Special Food, No.700 Changcheng Road, Qingdao 266109, China
*Corresponding author.
Qingli Yang, Ph. D., Tel: +86-186-6149-6099, Email: yql@qau.edu.cn or rice407@163.com

Apparatus and Characterization

For transmission electron microscope (TEM), The BCD complex samples (10 μL per sample) were dispersed in ultrapure water with $MgCl_2$ of 3 mM onto copper grids covered with carbon film. After drying at room temperature, TEM imaging was performed by HT7700 TEM (Hitachi Ltd., Japan). UV/Vis absorption spectra were measured on an Evolution 201 (Thermo Fisher, America). Fluorescence measurements were measured on a F-2700 fluorescence spectrophotometer (Hitachi Ltd., Japan). For agarose gel electrophoresis (AGE), the concentration of each sample was adjusted to 2 μM. The gel wells were loaded with 10 μL of sample. The gels were run with Mini-Sub Cell GT Cell (Bio-Rad, America) and Mini-PROTEAN Tetra (Bio-Rad, America), respectively. The gels of AGE were imaged under GelDoc EZ (Bio-Rad, America) and processed by Image Lab software (Bio-Rad, America). 10 μL of the sample were dropped onto a sheet of mica and dried in the air, and the atomic force microscope (AFM) measurements were carried out using a SPM-9700 (Shimadzu, Japan), operated in phase mode. Flow cytometer microscope was recorded by a Facs AriaIII (BD, America). Laser scanning confocal microscope (LSCM) was carried out on TCSsp5Ⅱ (Agilent, America). Dnamic light scattering (DLS) and Zeta potential were measured by Zetasizer Nano ZS (Malvern Ltd., UK). For Fourier Transform InfraRed (FT-IR) Spectrometer, 10 the μL of the sample was dropped in 0.13 g KBr powder. After being dried by an infrared sample lamp, the powder mixture was finely ground in an agate mortar, and then the powder was pressed into thin discs by a tablet press for scanning. FT-IR Spectrometer was obtained by NICOLET iS10 (Thermo Fisher, America).

Calculation of quantum fluorescence yield

Using reference method, quinine sulfate was used as reference substance to measure. A suitable concentration of quinine sulfate solution was prepared, and its UV absorption spectrum was measured, and its absorbance at 350 nm was recorded (A2) to ensure that the absorbance value was less than 0.05. Then, the fluorescence emission spectra in the range of 380 ~ 600 nm were obtained with 350 nm as excitation wavelength, and the fluorescence integral peak area (S2) was recorded. Similarly, a certain concentration of bCD solution was prepared, and the above steps were repeated to record its absorbance (A1) and fluorescence integral peak area (S1). The above values were substituted into formula (1), and the fluorescence quantum yield of carbon point was calculated to be 48.45%.

$$\mathrm{Y1} = \mathrm{Y2}\frac{\mathrm{S1A2n2}}{\mathrm{S2A1n1}} \qquad (1)$$

In Formula (1), digital 1 and 2 respectively represent the unknown substance to be measured and the reference standard substance, Y is the fluorescence quantum yield, S is the fluorescence peak area, A is the absorbance, and n refers to the refractive index of the solvent (The refractive index of water and 0.1M sulfuric acid solution are considered equal). In this study, quinine sulfate was selected as the standard reference substance, and its quantum yield (Y2) was 0.54 in 0.1 M sulfuric acid solution.

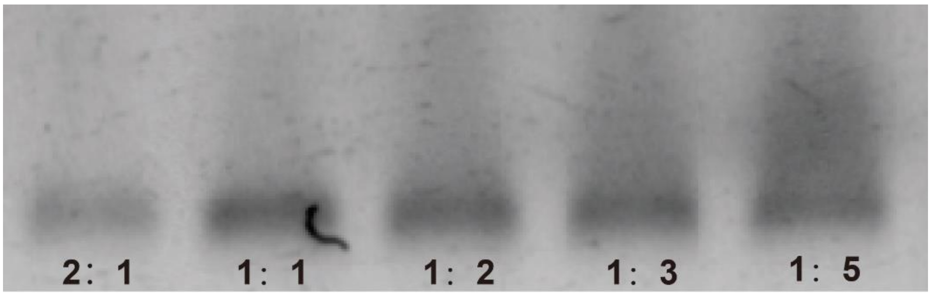

Figure S1. Molar ratio optimization for Td and bCD (the ratio in the picture is Td: bCD).

Table S1. Sequence of oligonucleotides used for self-assembly of Td1 and Td2.

Oligonucleotides	Sequence (5′–3′)
Apt1	GTG ACC GAT GAC AG TCC CTA CGG CGC TAA CCC CCC CAG TCC GTC CTC CCA GCC TCA CAC CGC CAC CGT GCT ACA AC [1]
Apt2	GTG ACC GAT GAC AG TCC CTA CGG CGC TAA CCT CCC AAC CGC TCC ACC CTG CCT CCG CCT CGC CAC CGT GCT ACA AC
T1	CTG TCA TCG GTC AC TGT AAG ATC GCG ACC ATT TGT TGA GCC TGG ACA GGT TCT ATG TGG CCA ATC AAT
T2-NH_2	NH_2-TCA TGG GAT ATC TAC GGT TCC TGT CCA GGC TCA ACT TAG ACT TCA GCT GGT TAT
T3-NH_2	NH_2 - TCC GTA GAT ATC CCA TGT TCG AGA GCA AGT GTA TGT TTT GAT TGG CCA CAT AGT
T4-NH_2	NH_2- TAT GGT CGC GAT CTT ACT TCA TAC ACT TGC TCT CGT TTA ACC AGC TGA AGT CTT
T2-des	Desthiobiotin-TCA TGG GAT ATC TAC GGT TCC TGT CCA GGC TCA ACT TAG ACT TCA GCT GGT TAT
T3-des	Desthiobiotin - TCC GTA GAT ATC CCA TGT TCG AGA GCA AGT GTA TGT TTT GAT TGG CCA CAT AGT
T4-des	Desthiobiotin - TAT GGT CGC GAT CTT ACT TCA TAC ACT TGC TCT CGT TTA ACC AGC TGA AGT CTT

*Design of the T1, T2, T3, and T4 oligonucleotides was adapted from previous report [2].

References

1. Chang, Y.-C.; Yang, C.-Y.; Sun, R.-L.; Cheng, Y.-F.; Kao, W.-C.; Yang, P.-C. Rapid single cell detection of Staphylococcus aureus by aptamer-conjugated gold nanoparticles. *Sci. Rep.* **2013**, *3*, 1863, doi:10.1038/srep01863.
2. Hong, C.-Y.; Zhang, X.-X.; Dai, C.-Y.; Wu, C.-Y.; Huang, Z.-Y. Highly sensitive detection of multiple antibiotics based on DNA tetrahedron nanostructure-functionalized magnetic beads. *Anal. Chim. Acta* **2020**, *1120*, 50–58, doi:10.1016/j.aca.2020.04.024.
